# Expression analysis of lymphocyte subsets and lymphocyte-to-monocyte ratio: reveling immunosuppression and chronic inflammation in breast cancer

**DOI:** 10.1007/s00432-023-05508-1

**Published:** 2024-01-23

**Authors:** Hao Zhang, Yan Li, Gang Liu, Xin Chen

**Affiliations:** 1 Department of Breast and Thyroid Surgery, The First Affiliated Hospital of Chongqing Medical University, Chongqing, China; 2 School of Public Health, Chongqing Medical University, Chongqing, China

**Keywords:** Lymphocyte subsets, Lymphocyte-to-monocyte ratio, Immunity, Chronic inflammation, Breast cancer, Immunosuppression, Diagnosis

## Abstract

**Objective:**

To explore the immune status and chronic inflammation of breast cancer patients, this study aims to analyze the diagnostic value of peripheral blood lymphocyte subsets (CD3^+^T, CD4^+^T, CD8^+^T, CD3^+^CD4^−^CD8^−^T, CD19^+^B, and NK cells) and lymphocyte-to-monocyte ratio (LMR) for breast cancer. Furthermore, it seeks to examine the correlation between these subsets and LMR with clinicopathological features.

**Methods:**

A total of 100 breast cancer patients were selected as the experimental group, while 55 patients with benign breast diseases were included in the control group. Statistical analysis, including the Wilcoxon test, Kruskal–Wallis test and the receiver operating characteristic curve, was employed to investigate the association between these serum indexes and the clinicopathological characteristics of the patients.

**Results:**

The levels of CD3^+^T cells, CD4^+^T cells, CD8^+^T cells, CD4^+^/CD8^+^ ratio, NK cells, CD3^+^CD4^−^CD8^−^T cells, and LMR were found to be related to the occurrence of breast cancer when analyzing data from patients with benign and malignant breast diseases. Among these biomarkers, CD3^+^T cells, CD4^+^T cells, CD4^+^/CD8^+^ ratio, CD3^+^CD4^−^CD8^−^T cells, and LMR were identified as independent risk factors for breast cancer development, and the AUCs were 0.760, 0.750, 0.598, 0.697, and 0.761 (P < 0.05), respectively. Furthermore, we observed varying degrees of differences in the expression of CD3^+^T cells, CD4^+^T cells, CD8^+^T cells, CD4^+^/CD8^+^ ratio, and LMR in lymph node metastasis, clinical staging, molecular typing, Ki-67 level (P < 0.05). However, statistical differences in histologic grade and pathology type were not found (P ≥ 0.05).

**Conclusion:**

Lymphocyte subsets and LMR reflect the immune status and chronic inflammation of the body, respectively. They have certain value in the diagnosis of benign and malignant breast diseases, and correlate with lymph node metastasis, clinical staging, molecular typing and other clinicopathological features of breast cancer. Therefore, monitoring the expression of lymphocyte subsets and LMR in the body may help the auxiliary diagnosis and condition analysis of breast cancer in the clinic.

## Introduction

Breast cancer has surpassed lung cancer as the malignant tumor with the highest incidence rate among women, with approximately 2.26 million new cases per year globally and 682,000 deaths, accounting for 15.5% of deaths from malignant tumors among women (Sung et al. 2021), which is yet another serious challenge for the global healthcare sector. The understanding of breast cancer has grown, revealing factors such as genetic predisposition, early menarche, late menopause, and infertility that contribute to its development. Furthermore, as the study of tumor immunity progresses, researchers have discovered that immune cells are vital in the progression of breast cancer, from the initial transformation of normal breast epithelial cells to the subsequent development of the disease (Goff and Danforth 2021). Lymphocytes, including CD3^+^T cells, CD4^+^T cells, CD8^+^T cells, CD3^+^CD4^−^CD8^−^T cells, CD19^+^B cells, and NK cells, are the main effector cells of the body’s immune response. CD3^+^T cells, the general term for T lymphocytes in the body, include CD4^+^T cells and CD8^+^T cells, which directly respond to the strength of cellular immunity. Studies have demonstrated that elevated levels of CD3^+^T cells in breast cancer patients are indicative of improved chemotherapy response rates and overall prognosis (Schmidt and Heimes 2021). CD4^+^T cells, as the hub of the immune system, secrete cytokines with anti-tumor effects and activate other immune effector cells (Kurozumi et al. 2017). CD4^+^T cell can be divided into Th1, Th2, regulatory T cells (Treg), etc. It is currently believed that Th1 cells mediate anti-tumor immunity, while Th2 and Treg cells have pro-tumorigenic effects due to their immunosuppressive properties (Ruffell et al. 2010; Zamarron and Chen 2011). Gu-Trantien et al. (Gu-Trantien et al. 2013) observed that CD4^+^T cells in the organism are an important immune-acting component in breast cancer, and that the stronger the immune response they generate, the better the disease-free survival (DFS) may be obtained. In a general way, high level of CD8^+^T cells is associated with better survival as they play a key role in the anti-tumor effects. CD8^+^T cells are activated to release granzyme and perforin, leading to the killing of target cells or induce apoptosis through the fas/fasl pathway. Additionally, CD8^+^T cells can differentiate into cytotoxic T-lymphocytes (CTLs), which directly exert anti-tumor effects (Mahmoud et al. 2011, 2012; Koretzky 2010). In an organism that normally executes an immune response, the majority of CD8^+^T cells are CTLs, while a few are suppressor T-lymphocytes (Ts) that inhibit the body’s immune process (Appay et al. 2008). The CD4^+^/CD8^+^ ratio reflects the balance in T-cell subsets. In patients with malignant tumors, this ratio can decrease or even reverse, indicating a disruption of immune homeostasis (Zacharakis et al. 2018). CD3^+^CD4^−^CD8^−^T cells, also known as DNT cells (double negative T cells), are a rare subset of T-cells of as yet unspecified origin. They have been found to play a role in inflammation, autoimmunity, and tumorigenesis (Wu et al. 2022). It has been suggested that DNT cells primarily exert their anti-tumor effects through various mechanisms such as secreting cytokines and the expression of tumor necrosis factor-associated apoptosis-inducing ligand (TRAIL) (Chen et al. 2019). Additionally, the reduced number of DNT cells may be associated with the decreased function of effector CD4^+^T cells with CTLs in tumor patients (Li et al. 2019). CD19^+^B cells, in contrast to the aforementioned lymphocytes, can convert CD4^+^T cells into Treg cells and enhance the immune-suppressive responses of tumor cells (Tadmor et al. 2011). NK cells are lymphocytes that have immunosurveillance and antitumor effects. They can directly kill tumor cells through nonspecific killing without needing specific sensitizers to activate them (Caligiuri 2008; Maxwell et al. 2018). However, tumor cells, in order to escape the immunocidal effect of the body, also produce immunosuppressive factors to inhibit the differentiation and proliferation of immune cells, researchers have detected decreased levels of immune cells and CD4^+^/CD8^+^ ratio in patients with lung, esophageal, colorectal, and gastric cancers (Chraa et al. 2019; Wang et al. 2021).

In addition, the role of chronic inflammation in tumor biology is gradually being recognized. Inflammatory response can release various inflammatory mediators that boost tumor microangiogenesis and hinder tumor cells senescence and death (Rius et al. 2008). Monocytes, as a type of inflammatory cells, which enter tumor tissues and differentiate into tumor associated macrophages (TAMs), form part of the inflammatory infiltration of tumors. It is currently believed that TAMs are predominantly classified as M2-type macrophages (Sica et al. 2006). TAMs secrete vascular endothelial growth factor (VEGF), platelet-derived growth factor (PDGF), epidermal growth factor (EGF), etc., which are involved in peritumor angiogenesis and promote tumor infiltration and metastasis (Pollard 2004). Additionally, TAMs release soluble factors that attenuate the cytotoxic function of T cell subsets, thereby suppressing the body’s anti-tumor immune response. High level of TAMs in breast cancer tissues is associated with high vascular density, a higher incidence of lymphatic metastasis, and poorer overall survival (OS) (Rius et al. 2008). In addition, monocytes themselves secrete pro-inflammatory cytokines, such as interleukins IL-1, IL-6, IL-10, and tumor necrosis factor (TNF-α) (Watari et al. 2014). Therefore, monocytes, as precursor components of macrophages, can be considered as a marker of the level of inflammatory response. Recently, many researchers have proposed combining the application of body immune function indexes and chronic inflammatory response indexes to obtain new predictive indexes like the lymphocyte-to-monocyte ratio (LMR). LMR not only reflects the level of anti-tumor immune response but also indicates the level of chronic inflammation in tumors, providing a more comprehensive assessment of the intra-organismal status of tumor patients. Studies have shown that high preoperative LMR is associated with a significant increase in time to recurrence (TTR) and OS in colon cancer patients, while patients with low LMR do not benefit much from adjuvant chemotherapy (Stotz et al. 2014). Additionally, Hu et al. (Hu et al. 2018) discovered a significant decrease in LMR among breast cancer patients. This decline was strongly linked to worse DFS and OS, particularly among Asian populations and TNBC patients.

The current clinical diagnosis of breast malignant tumors mainly relies on imaging and pathology examinations. However, imaging diagnosis is economically costly and has limitations such as lag. Pathologic diagnosis mainly consists of needle aspiration cytology and crude needle aspiration biopsy, which are invasive operations. Although pathology is considered the gold standard for diagnosis, some patients may experience psychological burden. Therefore, there is a need for another diagnostic method for early detection of breast malignant tumors. Currently, serological tests such as tumor markers have been successfully tried and widely used in clinics. Our research aims to explore another serological test index with diagnostic value. We will assess the predictive value of lymphocyte subsets and LMR on the occurrence, metastasis, and invasiveness of breast malignant tumors by integrating them with clinical features. This research will provide a new effective serological index for the early diagnosis of breast malignant tumors, offering a new diagnostic method to supplement imaging and pathology examinations.

## Patients and methods

### Patients and data collection

The test group for this study consisted of 120 patients with pathologically confirmed breast malignant tumors who attended the First Affiliated Hospital of Chongqing Medical University between August 2022 and July 2023. In the same period, 55 patients with benign breast lesions were selected as the control group. The studies involving human participants were reviewed and approved by the institutional ethics committee of the First Affiliated Hospital of Chongqing Medical University, and all experiments were performed in accordance with relevant guidelines and regulations. Pathological biopsy operations of all patients with breast malignant tumors were performed by experienced surgeons, and pathological results were determined by two experienced pathologists in our center. The inclusion criteria for the test group were as follows: (1) women aged 18–80 years, (2) histologically confirmed breast malignancy, and (3) no history of radiotherapy, chemotherapy, or targeted therapy prior to enrollment. The exclusion criteria were as follows: (1) prior anti-inflammatory drug therapy, (2) no history of acute or chronic inflammation, other neoplastic and hematological disorders, autoimmune diseases such as hyperthyroidism, rheumatoid arthritis, diabetes mellitus, and steroid medications, and (3) the presence of other major co-morbidities. Ultimately, 100 patients with breast malignant tumors were included in the study. The average age of the experimental group was 52.47 ± 10.40 years. Among the included patients, 52 cases (52.0%) were menopausal. Tumor sizes were as follows: 29 cases (29.0%) had a size of ≤ 2 cm, 57 cases (57.0%) had a size of > 2 cm and ≤ 5 cm, and 14 cases (14.0%) had a size of > 5 cm. Additionally, 68 cases (68.0%) had lymph node metastasis, and 5 cases (5.0%) had distant metastasis. According to the 8th edition of AJCC TNM staging, 57 cases (57.0%) were classified as ≤ TNM stage II, and 43 cases (43.0%) were classified as ≥ TNM stage III. Furthermore, 41 cases (41.0%) were hormone receptor-positive, 43 cases (43.0%) were HER-2 receptor-positive (regardless of hormone receptor expression), and 16 cases (16.0%) were triple-negative breast cancer (TNBC). According to the 2012 edition of the WHO classification of breast tumors, there were 8 cases (8.0%) of carcinoma in situ and 87 cases of invasive ductal carcinoma, including 4 cases (4.0%) of histologic grade I, 59 cases (59.0%) of grade II, 24 cases (24.0%) of grade III, and 5 cases (5.0%) of special type of invasive carcinoma. Based on a Ki-67 expression cut-off of ≤ 20%, Ki-67 low expression (Ki-67 ≤ 20%) in 36 cases (36.0%), and high expression (Ki-67 > 20%) in 64 cases (64.0%). In the control group of 55 women with benign breast diseases, the average age was 49.18 ± 11.14 years. Among them, 26 cases (47.3%) were menopausal. The distribution of tumor sizes in the control group was as follows: 21 cases (38.2%) had a size of ≤ 2 cm, 24 cases (43.6%) had a size of > 2 cm and ≤ 5 cm, and 10 cases (18.2%) had a size of > 5 cm. Furthermore, the differences in age, menstrual status, and tumor size between the two groups were not statistically significant (P > 0.05) and were comparable (Table 1).

**Table 1:** Baseline information table and univariate analysis

	Total (n = 155)	Benign (n = 55)	Malignant (n = 100)	P-value
Patients	155	55	100	–
Age	51.30 ± 10.75	49.18 ± 11.14	52.47 ± 10.40	0.068
Menopausal status				0.573
Menopausal	78 (50.3%)	26 (47.3%)	52 (52.0%)	
Non-menopausal	77 (49.7%)	29 (52.7%)	48 (48.0%)	
Tumor size				0.576
≤ 2 cm	50 (32.3%)	21 (38.2%)	29 (29.0%)	
> 2 cm and ≤ 5 cm	81 (52.2%)	24 (43.6%)	57 (57.0%)	
> 5 cm	24 (15.5%)	10 (18.2%)	14 (14.0%)	
CD3^+^	1202.00 (963.00,1462.00)	1404.00 (1159.00,1701.00)	1089.00 (838.50,1342.75)	< 0.001*
CD3^+^CD4^+^	716.00 (561.00,890.00)	816.00 (669.00,1052.00)	636.50 (470.75,763.00)	< 0.001*
CD3^+^CD8^+^	39.40 (321.00,506.00)	429.00 (360.00,559.00)	384.00 (298.50,459.75)	0.009*
CD4^+^/CD8^+^	1.72 (1.37,2.23)	1.80 (1.42,2.61)	1.60 (1.30,2.09)	0.043*
CD3^+^CD4^−^CD8^−^	74.00 (50.00,116.00)	96.00 (69.00,178.00)	66.00 (43.25,96.00)	< 0.001*
CD19^+^B	213.00 (166.00,293.00)	224.00 (180.00,323.00)	210.00 (161.25,287.00)	0.068
NK	214.00 (132.00,298.00)	251.00 (174.00,347.00)	205.00 (122.25,264.25)	0.019*
LMR	4.57 (3.46,5.75)	5.46 (4.54,6.52)	3.96 (3.07,5.08)	< 0.001*

*Values in bold represent statistically significant results (P < 0.05)

### Detection of lymphocyte subsets

Before treatment, all study subjects were fasted and 3 ml of peripheral venous blood was drawn into a disposable blood collection tube. The blood was then immediately sent for examination and stored at room temperature after anticoagulation with EDTA-K2, while avoiding shaking. The flow cytometry steps involved adding 20 μL of murine anti-human CD3, CD4, CD8, CD16, CD19, CD56 fluorescently labeled monoclonal antibody to the bottom of the flow tube, thoroughly mixing it, and allowing it to label for 30 min at a temperature range of 20–25 °C, all while avoiding exposure to light. Following that, 3 mL of PBS was added for washing purposes. After centrifugation at 1500 r/min for 5 min, an additional 1 mL of PBS was added for further mixing. Subsequently, CD3^+^T cells, CD3^+^CD4^+^T cells, CD3^+^CD8^+^T cells, CD3^+^CD4^−^CD8^−^T cells, CD3^−^CD16^+^CD56^+^ NK cells, and CD19^+^B cells were detected using the flow cytometer. The mouse monoclonal antibodies and flow cytometers were purchased from BD, USA, and were utilized as per the provided instructions.

### Blood tests

5 mL of venous blood was drawn on an empty stomach and placed in an anticoagulation tube. The blood was then subjected to centrifugation at a centrifugal rate of 1500 r/min and a centrifugal radius of 15 cm for 10 min. Using a Mindray BC5180 automatic hematology analyzer and accompanying reagents, the levels of white blood cells, lymphocyte, and monocyte were detected. The test was repeated 3 times, and the average of the 3 results was taken as the final examination result. Subsequently, the lymphocyte and monocyte counts were collected, and the lymphocyte-to-monocyte ratio (LMR) was calculated, defined as the ratio of lymphocyte count to monocyte count.

### Statistical analysis

The clinical characteristics of patients in the two groups were expressed as frequencies or descriptive analysis. If the quantitative information satisfied normal distribution, it was expressed as mean ± standard deviation, and comparisons between two groups were conducted using the independent samples t-test. On the other hand, if the quantitative information was skewed, it was expressed as M (P25, P75), and comparisons between two groups were made using the Wilcoxon (Mann–Whitney U) test. For comparisons between multiple groups, the Kruskal–Wallis (KW test) was utilized. Qualitative information was expressed as n (%), and comparisons between groups were performed using the chi-square test or the KW test. Multifactorial analysis was conducted using binary logistic regression. Additionally, the diagnostic value of the above indexes for breast malignant tumors was analyzed using the receiver operating characteristic curve (ROC curve). Statistical analysis was performed using SPSS 25.0.

## Result

### Univariate analysis reveals possible factors in the development of breast malignancy

The results showed that age, menstrual status, and tumor size did not demonstrate statistical significance in both the benign and malignant lesion groups (P ≥ 0.05). On the other hand, statistically significant differences were observed in CD3^+^T, CD4^+^T, CD8^+^T, CD4^+^/CD8^+^, DNT, NK, and LMR between the two groups of patients (P < 0.05) (Table 1).

### Multifactorial analysis reveals independent risk factors for the development of breast malignancy

Indicators with statistically significant results in the univariate analysis were included as independent variables in a multifactorial logistic regression model, which showed that CD3^+^T, CD4^+^T, CD4^+^/CD8^+^, DNT, and LMR were independent risk factors for the development of malignant tumors in the breast (P = 0.001, < 0.001, = 0.043, = 0.025, < 0.001) (Table 2).

**Table 2:** Comparison of independent risk factors for breast malignancy

Indicators	B	SR	Wald	P-value	OR	OR 95% CI	
						Lower limit	Upper limit
CD3^+^	− 0.002	0.001	10.257	0.001	0.998	0.996	0.999
CD3^+^CD4^+^	− 0.004	0.001	19.251	< 0.001	0.996	0.994	0.998
CD4^+^/CD8^+^	− 0.631	0.311	4.109	0.043	0.532	0.289	0.979
CD3^+^CD4^−^CD8^−^	− 0.007	0.003	4.982	0.025	0.993	0.987	0.999
LMR	− 0.543	0.153	12.540	< 0.001	0.581	0.430	0.785

### ROC curve reveals the diagnostic value of each index for breast malignant tumors

**Fig. 1:**
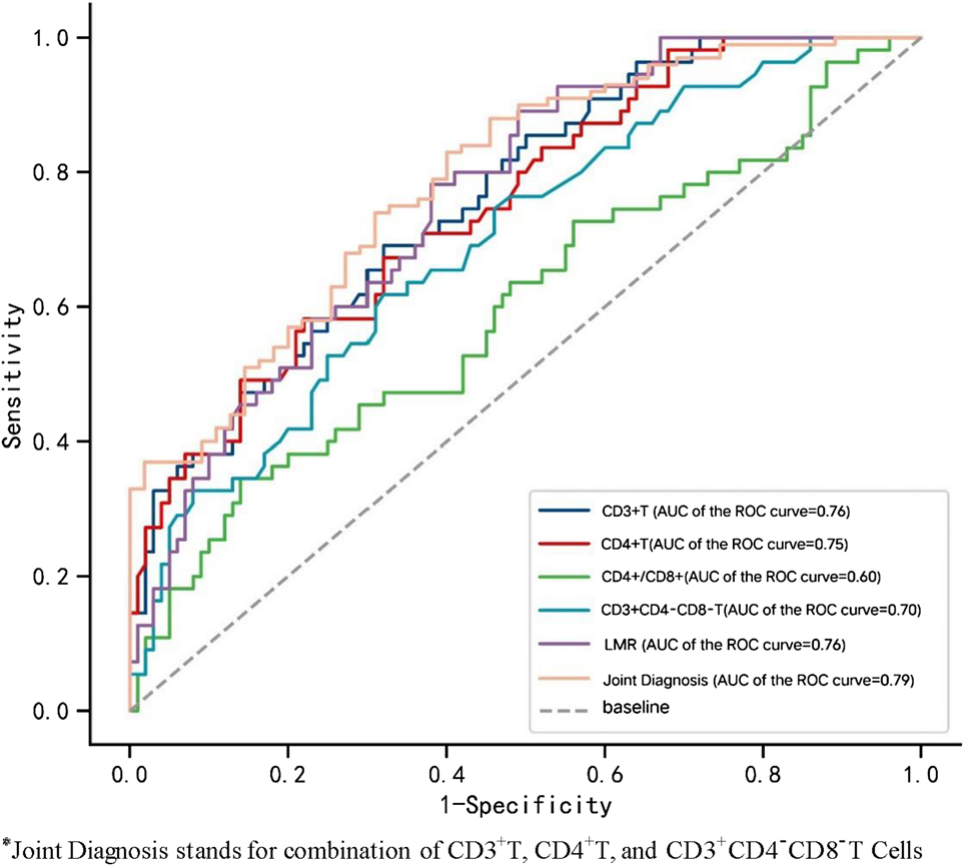
The ROC curve graph of lymphocyte subsets and LMR in breast cancer and benign breast disease patients

The results of ROC curve analysis demonstrated that the AUCs for CD3^+^T, CD4^+^T, CD4^+^/CD8^+^, DNT, and LMR were 0.760 (95% CI 0.684–0.836), 0.750 (95% CI 0.672–0.828), 0.598 (95% CI 0.503–0.694), 0.697 (95% CI 0.612–0.781), and 0.761 (95% CI 0.686–0.835), respectively. Sensitivities for these indicators were 69.1%, 58.2%, 34.5%, 61.8%, and 78.2%, while specificities were 68.0%, 78.0%, 86.0%, 68.0%, and 62.0%. Moreover, when T-lymphocyte subsets CD3^+^T, CD4^+^T, and DNT were combined for diagnostic purposes, the resulting AUC was 0.790 (95% CI 0.717–0.862), with a sensitivity of 74.0% and specificity of 69.1% (Table 3; Fig. 1). Hence, serum levels of CD3^+^T, CD4^+^T, CD4^+^/CD8^+^, DNT, and LMR have significant associations with the development of breast malignant tumors, demonstrating their diagnostic value. Furthermore, the combined application of these indicators can further enhance their diagnostic value.

**Table 3:** ROC analysis of lymphocyte subsets and LMR in differentiating breast cancer from benign control group

Indicators	AUC	Sensitivity	Specificity	Yoden Index	Threshold
CD3^+^	0.760	0.691	0.680	0.371	1248.000
CD3^+^CD4^+^	0.750	0.582	0.780	0.362	782.000
CD4^+^/CD8^+^	0.598	0.345	0.860	0.205	2.300
CD3^+^CD4^−^CD8^−^	0.697	0.618	0.680	0.298	83.000
LMR	0.761	0.782	0.620	0.402	4.511
Joint diagnosis	0.790	0.740	0.691	0.431	0.640

### Relationship between lymphocyte subsets and LMR with clinical features of breast malignancy

We further analyzed 100 patients from the breast malignancy group to find associations between lymphocyte subsets and LMR with clinical features of breast malignancy. Compared with the group without lymphatic metastasis, the group with lymphatic metastasis exhibited a significant decrease in the levels of CD3^+^T, CD4^+^T, DNT, and LMR (P < 0.05). However, no statistically significant relationship was found between other indexes and lymphatic metastasis (P ≥ 0.05) (Table 4). We also distinguished whether there was any difference in lymphocyte subsets and LMR levels between different TNM stages. In patients with early stage (≤ stage II) compared to patients with advanced breast malignancy (≥ stage III), statistically significant findings were observed. Specifically, there was a significant increase in CD8^+^T, and a significant decrease in CD4^+^/CD8^+^ and LMR (P < 0.05). However, other indicators did not show statistically significant differences in relation to clinical stage (P ≥ 0.05) (Table 5).

**Table 4:** Lymphocyte subsets and LMR associated with lymph node metastasis

Indicators	Presence of lymph node metastasis		P-value
	No lymph node metastasis group (n = 32)	Group with lymph node metastasis (n = 68)	
CD3^+^	1177.50 (1038.75,1392.75)	1022.00 (793.00,1293.00)	**0.030**
CD3^+^CD4^+^	715.00 (607.00,762.50)	591.00 (444.00,760.00)	**0.014**
CD3^+^CD8^+^	395.50 (317.00,462.75)	380.00 (286.00,445.00)	0.346
CD4^+^/CD8^+^	1.86 (1.32,2.22)	1.59 (1.28,2.00)	0.113
CD3^+^CD4^−^CD8^−^	76.50 (61.50,96.00)	61.00 (36.50,99.25)	**0.043**
CD19^+^B	193.50 (166.25,327.75)	213.00 (150.50,261.50)	0.329
NK	221.50 (148.00,284.50)	196.00 (100.25,260.25)	0.085
LMR	4.71 (3.25,5.72)	3.7 (2.93,4.78)	**0.037**

Values in bold represent statistically significant results (P < 0.05)

**Table 5:** Lymphocyte subsets and LMR associated with TNM staging

Indicators	TNM Staging		P-value
	Stage 0–II (n = 57)	Stage III–IV (n = 43)	
CD3^+^	1067.00 (859.00,1243.50)	1105.50 (831.00,1494.00)	0.145
CD3^+^CD4 ^+^	643.00 (493.00,751.50)	630.00 (463.25,861.00)	0.484
CD3^+^CD8^+^	360.00 (275.50,414.00)	399.50 (339.00,540.00)	**0.002**
CD4^+^/CD8^+^	1.84 (1.37,2.19)	1.45 (1.20,1.86)	**0.015**
CD3^+^CD4^−^CD8^−^	71.00 (43.50,95.00)	63.00 (40.00,102.00)	0.805
CD19^+^B	193.00 (156.00,291.00)	214.00 (166.00,288.00)	0.638
NK	214.00 (132.00,279.00)	203.00 (94.00,257.00)	0.356
LMR	4.44 (3.26,5.52)	3.49 (2.77,4.42)	**0.005**

Values in bold represent statistically significant results (P < 0.05)

### Relationship between lymphocyte subsets and LMR with molecular typing

In this study, 100 patients with breast malignant tumors were divided into three groups: the HR (+) group with 41 cases, the HER-2 (+) group with 43 cases, and the TNBC group with 16 cases. Statistical analysis revealed that there were no statistically significant differences in the levels of CD4^+^/CD8^+^, DNT, NK, and LMR among the three groups (P ≥ 0.05). However, significant differences were found in CD3^+^T, CD4^+^T, and CD8^+^T between the groups (P < 0.05). Specifically, CD4^+^T was significantly lower in the HER-2 (+) group compared to the HR (+) group and the TNBC group. CD3^+ ^and CD8^+^T were significantly lower in the HER-2 (+) group compared to the TNBC group. It is worth noting that there were no significant differences in the above indexes between the HR (+) and the TNBC groups (P ≥ 0.05) (Table 6).

**Table 6:** Lymphocyte subsets and LMR associated with molecular typing

Indicators	Hormone receptor-positive type (n = 41)	HER-2 receptor-positive type (n = 43)
CD3^+^	1137.00 (984.00,1365.00)	931.00 (755.00,1176.00)
CD3^+^CD4^+^	681.00 (575.50,785.50)	548.00 (444.00,716.00)
CD3^+^CD8^+^	393.00 (289.50,454.50)	333.00 (286.00,413.00)
CD4^+^/CD8^+^	1.63 (1.28,2.27)	1.59 (1.38,2.00)
CD3^+^CD4^−^CD8^−^	74.00 (48.50,112.50)	57.00 (40.00,87.00)
CD19^+^B	228.00 (172.00,312.00)	185.00 (132.00,245.00)
NK	213.00 (137.50,263.50)	207.00 (97.00,271.00)
LMR	3.97 (3.01,5.08)	3.69 (2.73,4.80)

Indicators	Triple negative type (n = 16)	P-value	P1*	P2*	P3*
CD3^+^	1333.00 (897.00,1497.00)	**0.004**	0.076	**0.006**	0.460
CD3^+^CD4^+^	754.00 (611.00,921.00)	**0.003**	**0.025**	**0.007**	0.879
CD3^+^CD8^+^	445.00 (368.00,526.00)	**0.017**	0.534	**0.014**	0.212
CD4^+^/CD8^+^	1.61 (1.31,1.88)	0.690	–	–	–
CD3^+^CD4^−^CD8^−^	74.50 (57.25,99.25)	0.237	–	–	–
CD19^+^B	202.50 (161.25,277.25)	0.050	–	–	–
NK	203.00 (122.00,340.00)	0.856	–	–	–
LMR	4.68 (3.67,6.36)	0.113	–	–	–

Values in bold represent statistically significant results (P < 0.05)

*P1, Difference in lymphocyte subsets and LMR between hormone receptor-positive and HER-2 receptor-positive types. *P2, Difference in lymphocyte subsets and LMR between HER-2 receptor-positive and triple-negative types. *P3, Difference in lymphocyte subsets and LMR between hormone receptor-positive and triple-negative types

### Relationship between lymphocyte subsets and LMR with Ki-67 level

Based on the Ki-67 ≤ 20% threshold, 100 patients with breast malignant tumors were categorized into Ki-67 low-expression and high-expression groups. Specifically, there were 36 cases in the low-expression group and 64 cases in the high-expression group. The results of the statistical analysis indicated that the low-expression group exhibited significantly higher expression levels of CD3^+^T, CD4^+^T, and CD8^+^T compared to the high-expression group (P < 0.05). Conversely, no statistically significant differences were observed between the two groups in terms of CD4^+^/CD8^+^, DNT, CD19^+^B, NK, and LMR (P ≥ 0.05) (Table 7).

**Table 7:** Lymphocyte subsets and LMR associated with Ki-67 expression

Indicators	Ki-67		P-value
	Low expression (n = 36)	High expression (n = 64)	
CD3^+^	1220.00 (979.75,1472.25)	1038.00 (779.00,1231.00)	**0.004**
CD3^+^CD4^+^	727.00 (568.25,901.25)	611.00 (428.00,728.00)	**0.005**
CD3^+^CD8^+^	397.50 (331.25,529.25)	368.00 (280.00,430.00)	**0.042**
CD4^+^/CD8^+^	1.61 (1.32,2.22)	1.60 (1.28,2.01)	0.638
CD3^+^CD4^−^CD8^−^	74.50 (44.50,100.75)	63.00 (39.50,90.00)	0.432
CD19^+^B	214.50 (161.25,309.50)	204.00 (159.75,277.00)	0.573
NK	196.00 (135.75,247.75)	216.00 (118.75,271.00)	0.566
LMR	4.35 (3.09,5.47)	3.90 (3.03,4.80)	0.291

Values in bold represent statistically significant results (P < 0.05)

### Relationship between lymphocyte subsets and LMR with histologic grading and pathology type

We divided 100 patients with breast malignant tumors into three groups based on the pathological results: ductal carcinoma in situ group, special type invasive carcinoma group, and invasive ductal carcinoma group. The invasive ductal carcinoma group was further divided into histological grade I group, histological grade II group, and histological grade III group, according to the WHO breast tumor classification (2012 version) standard. However, statistical analysis did not show significant differences in the expression levels of the indicators between these five groups (P ≥ 0.05) (Table 8).

**Table 8:** Lymphocyte subsets and LMR associated with histologic grading and pathology type

Indicators	Histopathological grade (n = 86)			DCIS (n = 9)	Infiltrative non-specific cancer (n = 5)	P-value
	I (n = 3)	II (n = 59)	III (n = 24)			
CD3^+^	1535.00 (1035.00,1552.50)	1061.00 (823.00,1305.00)	1107.00 (853.00,1431.50)	1137.00 (1014.00,1322.00)	972.0 (669.00,1395.50)	0.435
CD3^+^CD4^+^	743.00 (415.00,963.50)	627.00 (465.00,749.00)	674.50 (476.50,851.25)	728.00 (602.00,846.00)	628.00 (373.50,788.00)	0.527
CD3^+^CD8^+^	367.00 (286.00,556.50)	393.00 (298.00,466.00)	374.00 (301.00,490.00)	395.00 (270.00,431.50)	341.00 (256.50,573.50)	0.991
CD4^+^/CD8^+^	1.45 (1.00,2.34)	1.53 (1.27,2.15)	1.85 (1.45,2.00)	1.78 (1.41,2.41)	1.37 (1.26,1.92)	0.612
CD3^+^CD4^−^CD8^−^	88.00 (63.00,210.50)	63.00 (40.00,102.00)	68.50 (54.00,116.5)	75.00 (30.00,102.00)	55.00 (22.00,66.50)	0.286
CD19^+^B	246.00 (148.00,294.00)	193.00 (158.00,268.00)	235.00 (181.25,300.00)	170.00 (143.00,245.50)	255.00 (158.00,295.00)	0.554
NK	165.00 (92.00,204.50)	213.00 (122.00,271.00)	205.00 (125.75,265.00)	176.00 (127.00,257.00)	123.00 (90.50,318.50)	0.925
LMR	5.08 (4.26,5.25)	3.78 (2.91,5.08)	4.27 (3.06,4.79)	3.80 (3.18,4.78)	4.73 (3.90,5.55)	0.753

## Discussion

More attention has been paid to the tumor microenvironment of breast cancer due to the progress of molecular immunology. Tumor infiltrating lymphocytes (TILs) have emerged as a significant area of interest in this regard. However, the current detection methods for TILs are complex and expensive, limiting their widespread implementation. Ruffell et al. (2012) discovered that TILs in solid tumors primarily consist of CD3^+^T-lymphocytes, with a small population of B-lymphocytes and NK cells. Conversely, lymphocytes in peripheral blood, which serve as the main source of TILs, can indirectly reflect the TILs level in patients. This alternative approach offers several advantages, including simplicity, cost-effectiveness, and the ability to dynamically monitor the patient’s immune response at different disease stages, presenting promising clinical potential. Furthermore, our research also explores the chronic inflammatory response caused by tumors, in addition to focusing on the patient’s immune status. Interestingly, we found that the relationship between lymphocytes levels and chronic inflammation in breast cancer patients may have intriguing implications.

Although decreased levels of CD3^+^T cells, CD4^+^T cells, CD8^+^T cells, NK cells and CD4^+^/CD8^+^ ratio have been detected in the peripheral blood of patients with malignant tumors such as lung, esophageal, colorectal, and gastric cancers, the degree of immune suppression may also be inconsistent across tumors. In this study, we demonstrated that significantly reduced levels of CD3^+^T cells, CD4^+^T cells, CD8^+^T cells, DNT cells, NK cells, CD4^+^/CD8^+^ ratio, and LMR were detected in the peripheral blood of patients with breast cancer relative to patients with benign breast lesions. Reduced CD3^+^T cells, CD4^+^T cells, DNT cells, CD4/CD8 ratio, and LMR were considered to be independent risk factors for the development of breast cancer. CD3^+^T cells, which represent the overall level of cellular immunity in the body, were found to be significantly decreased in breast cancer patients. This indicates the existence of immunosuppression in patients with breast cancer, potentially related to tumorigenesis. On the other hand, the levels of CD4^+^T cells, CD8^+^T cells, CD4^+^/CD8^+^ ratio, DNT cells, and NK cells in breast cancer patients have been reduced to different degrees within the lymphocyte subsets. This reduction can be attributed to the tumor releasing immunosuppressive factors, such as TGF-β, IL-10, during its growth. These factors inhibit the differentiation and proliferation of lymphocytes, leading to an imbalance of Th1/Th2 within the CD4^+^T cells, favoring Th2 cells, and promoting the generation of Ts cells. Consequently, the number and activity of lymphocytes with anti-tumor effects are suppressed to varying degrees (Janneh et al. 2009; Yuan et al. 2013). In addition, the increase in Ts and Th2 cells inhibits the formation and maturation of CD4^+^T cells and other immune cells, resulting in a decrease in CD4^+^T cells and an increase in CD8^+^T cell reactively. The predominance of Ts cells in the CD8^+^T cells ultimately leads to a decrease or even inversion of the CD4^+^/CD8^+^ ratio, indicating dysfunction in the body’s immune function and compromised anti-tumor efficacy (Mamessier et al. 2011; Trédan et al. 2013). Studies have shown that a lower CD4^+^/CD8^+^ ratio is associated with a higher recurrence and metastasis rate of tumors, which has implications for poor prognosis (Xu et al. 2017). Recent studies have found that DNT cells exert anti-tumor effects in xenograft models of leukemia, lung cancer, and pancreatic cancer. (Lee, et al. 2018; Yao et al. 2019; Lu et al. 2019). Our research has also revealed a significant decrease in the number of DNT cells in breast cancer patients. This suggests that the reduction of DNT cells may play a promoting role in the development of breast cancer. Additionally, researchers have discovered that the addition of allogeneic DNT cells in vitro models does not lead to significant rejection (Lee et al. 2018), indicating the potential of DNT cells in anti-tumor immunotherapy. The decrease in LMR observed in breast cancer patients is partly due to tumor-induced reduction of lymphocytes caused by tumor immunosuppression as mentioned above and partly due to an increase in monocyte production resulting from inflammation. During the process of tumor transformation to malignancy, the body’s repair mechanism is disrupted, leading to chronic inflammation. At the same time, tumor cells consume a significant amount of oxygen during their growth, resulting in a localized hypoxic environment that induces hypoxic stress in the body. This state of stress triggers the release of mononuclear chemokine ligand 2 (CCL2). As a consequence, numerous monocytes are recruited from the vasculature to the tumor microenvironment, where they differentiate into TAMs (Hagemann et al. 2006). Moreover, the stress induces Treg production that can both enhance tumor angiogenesis and inhibit anti-tumor responses (Facciabene et al. 2011). TAMs produce a variety of pro-angiogenic growth factors and proteases, such as VEGF, MMP-9, and uPA, to promote angiogenesis and oxygenation of tumors. Furthermore, TAMs restrain the proliferation of T-lymphocytes by releasing mediators such as prostaglandins and IL-10. The increased survival rate is associated with low level of TAMs in the tumor microenvironment (Sica et al. 2008). Wilcox et al. (Wilcox et al. 2009) also found that monocytes and their progeny in the tumor microenvironment are able to stimulate Treg proliferation by expressing PD-L1. Consequently, chronic inflammation can lead to immunosuppression, resulting in tumor escape. Statistically, chronic inflammation has been found to be related to around 15%-20% of tumor pathogenesis in various cancer types, such as hepatitis B virus and liver cancer, helicobacter pylori and gastric cancer, and inflammatory bowel disease and colon cancer (Allavena et al. 2008; Mantovani et al. 2008), highlighting its role in promoting tumorigenesis. Moreover, NSAIDs can reduce the production of inflammatory mediators such as prostaglandins and thromboxane A2, thus reducing the inflammatory response to exert a cancer-suppressive effect (Balkwill and Coussens 2004). Some studies have shown that taking NSAIDs can reduce the risk of colon cancer by 40–50% compared with the control group (Rodríguez and Huerta-Alvarev 2001). This have further illustrated the role of chronic inflammation in promoting tumorigenesis (Greten and Grivennikov 2019). As a result, Hal Dvorak says, "Tumors are wounds that don’t heal." (Dvorak 2015). Therefore, controlling chronic inflammation through some interventions like NSAIDs has shown promise in reducing the risk of certain cancers.

Currently, clinical factors such as tumor stage, lymph node metastasis, and molecular typing impact the severity and prognosis of breast cancer. To establish a better understanding of the relationship between breast cancer, immune function, and chronic inflammation, we conducted additional analyses.

Lymph node metastasis, being the most common metastatic component of breast cancer, has been shown to affect the prognosis of patients. Studies indicate that compared with breast cancer patients without lymph node metastasis, patients with lymph node metastasis have a higher degree of malignancy and a significantly lower survival rate (To et al. 2020). Consequently, immune escape is more severe in these patients. In this study, we observed that patients with lymphatic metastasis exhibited significantly lower levels of CD3^+^T cells, CD4^+^T cells, and CD3^+^CD4^−^CD8^−^T cells. Particularly a notable decrease in the main effector cells, CD4^+^T cells. This decrease in CD4^+^T cells is likely to lead to a significant reduction in the tumor-eliminating effect, consequently facilitating the tumor cells evasion from the primary site. Moreover, we noted a significant decrease in the LMR among patients with lymph node metastasis. We hypothesize that this reduced LMR is associated with an intensified chronic inflammatory response in these patients. NF-kB is activated by chronic inflammation, leading to an increase in the metastatic potential of cancer cells. This occurs through the promotion of angiogenesis to provide oxygen to cancer cells, as well as by stimulating proliferation and inhibiting apoptosis (Cao and Prescott 2002). In a study comparing mice with NF-kB activation to those with NF-kB inactivation, comparable levels of cell proliferation and atypical hyperplasia were observed at the tumor initiation stage. However, as mice mature and tumors progress, tumor cells in the mice with activated NF-kB exhibited significantly proliferative activity (Pikarsky et al. 2004). Additionally, COX-2 also secretes substances such as VEGF, PGE2 and TXA2, which promote tumor cells proliferation and metastasis in a manner similar to NF-kB activation (Ono 2008). This mechanism may primarily impact on the tumor progression rather than its initiation or early stages. We further analyzed the relationship between clinical stage and the levels of lymphocyte subsets and LMR. Patients with stage III-IV may observe an increase in CD8^+^T cells and experience a significant decrease CD4^+^/CD8^+^ ratio and LMR when compared to patients with stage I–II. This could be due to the continuous release of immunosuppressive molecules during tumor development, which leads to the dominance of lymphocytes with an inhibitory effect and exacerbates the inflammatory response. The immune damage becomes more severe as the tumor stage progresses (Chen et al. 2014). Ts cells phenotyped as CD8^+^ are expressed in large numbers, while Th cells activities are reduced. The dominance of Ts and Treg cells with pro-tumorigenic effects aids in immune escape by tumor cells. Consequently, patients with advanced breast cancer may witness an increase in CD8^+^T cells and experience a significant decrease or even reversal of the CD4^+^/CD8^+^ ratio. However, there is no statistically significant difference in CD4^+^T cells level between the two groups. It is hypothesized that although Th1 cells are reduced in advanced patients, Treg cells are increased within the CD4^+^ subset due to the hypoxic environment and chronic inflammation (Hagemann et al. 2006), resulting in no significant change in overall CD4^+^T cells levels. This is supported by a study by Burugu et al. (2017) who found that patients with advanced breast cancer had predominantly mesenchymal infiltrating CD4^+^T cells with Treg and Th2. Treg in CD4^+^T cells could reach 20–40% of total CD4^+^T subset. However, in this study, the level or ratio of Treg and Ts cells in peripheral blood was not tested separately. Future studies cananalyze the composition of CD4^+^T and CD8^+^T cells internal lymphocytes in patients with breast cancer.

In this study, it was found that HER-2 (+) patients had significantly lower levels of CD3^+^T and CD8^+^T cells compared to TNBC patients. Additionally, the level of CD4^+^T cells was lower in HER-2 (+) patients compared to both TNBC and HR (+) patients. Consequently, the researchers conclude that HER-2 (+) patients experience the most apparent immunosuppression. The overexpression of HER-2, which is a proto-oncogene with tyrosine kinase activity, promotes tumor cell proliferation, inhibits apoptosis, and facilitates tumor angiogenesis, thus increasing tumor invasiveness. Datta et al. (2016) reported an increased proportion of Treg cells in HER-2 (+) patients with advanced breast cancer, and this proportion decreased after trastuzumab treatment. Similarly, Liu et al. (2011) observed that Treg cells were significantly elevated in HER-2 (+) patients, and the higher CTL/Treg ratio in the paraneoplastic tissues was associated with better progression-free survival (PFS) and OS. Hence, while blocking the HER-2 pathway, using Treg cells expression as a target to counteract the cancer-associated immunosuppression is a potential therapeutic approach that may improve the prognosis of HER-2 (+) patients. Furthermore, assessing the levels of Treg and CD4^+^T cells in HER-2 (+) patients can be valuable for monitoring the condition of breast cancer patients and predicting the efficacy of anti-HER-2 treatment.

Ki-67 is a nuclear protein discovered by Gerdes et al. (1983) as a cell proliferation marker, which responds to the proliferative activity of tumor cells. In this study, we found that the levels of CD3^+^T, CD4^+^T and CD8^+^T cells were significantly reduced in the peripheral blood of breast cancer patients with high Ki-67 expression. This finding confirms that immunosuppressive factors are constantly released during tumor proliferation, which is consistent with earlier descriptions. The degree of decrease in the level of immune effector cells is more pronounced with stronger proliferative activity of the tumor cells. It is worth noting that the overall survival rate of breast cancer has been reported to be correlated with histologic grade. High histologic grade is identified as a risk factor for recurrent metastasis of breast cancer (Bianchini and Gianni 2014). Nevertheless, our study did not find a statistical relationship between lymphocyte subsets and LMR with histologic grade and pathology type. This lack of correlation may be attributed to the insufficient sample size or the selection bias resulting from the inclusion of a high percentage of patients with histologic grades II and III. Notably, this high percentage may be due to the heavy tumor load of most of the patients enrolled in our study.

Recent studies have found that CD19^+^B cells promote tumor development in vitro models. Inoue et al. (2006) showed that B-cells depletion may enhance the anti-tumor immune response in a B-cells-deficient mouse model. CD19^+^B cells are thought to promote breast cancer development and metastasis through the secretion of IL-10 and induction of the conversion of CD4^+^T cells to Treg in the mouse models (Olkhanud et al. 2011; Gray et al. 2007). However, our study has not found a significant elevation in the expression level of CD19^+^B cells in breast cancer patients. Additionally, there is no significant correlation between CD19^+^B cells and clinical features of breast cancer patients. Yang et al. (Yang et al. 2019) found that CD3^+^T and CD4^+^T cells are independent predictors of PFS and OS in metastatic breast cancer patients with different molecular phenotypes, but no predictive value is found for CD19^+^B cells in relation to prognosis, nor any association between CD19^+^B cells and clinical factors in breast cancer. However, considering the potential role of CD19^+^B cells in promoting Treg, future research can further measure Treg level to elucidate the inherent connection between CD19^+^B cells and Treg. These efforts may uncover the potential reasons for discrepancies in conclusions about CD19^+^B cells obtained from experimental models and clinical research.

This study indicates the formation of a closed loop connecting breast cancer, chronic inflammation, and the immune status of the body. Although malignant tumor primarily originates from genetic mutations, but it prompts changes in the body’s microenvironment. These changes then trigger chronic inflammation, which can further promote tumor progression. Both the tumor cells and chronic inflammation simultaneously cause damage to the body’s immune function through various pathways. And suppression of immune function correspondingly accelerates the progression of tumors and diminishes the elimination of inflammation. Blocking any of the pathways on this closed loop may be a strategy to improve the therapeutic efficacy and prognosis of breast cancer patients. In particular, the development of bioimmunology highlights cellular immunotherapy as an important direction for breast cancer patients who respond poorly to conventional therapy. Nevertheless, there are limitations to our study. Firstly, this is a single-center study with a small sample size. To confirm our current conclusions, a large-sample multicenter clinical study is needed. Secondly, this study only collected venous blood once during the hospitalization of the patients for examination. However, it is important to note that the immune damage caused by chronic inflammation and tumors is a long-lasting process. Therefore, a dynamic follow-up is required to clearly observe this chronic damage process. Furthermore, long-term follow-up can also collect the survival status of the patients, thus aiding in determining the prognostic value of lymphocyte subsets and LMR in breast cancer patients. This work can serve as a foundation for further diagnostic studies.

## Conclusion

The results of this paper revealed the role of immunosuppression and chronic inflammatory state in the transformation of normal epithelial cells of the breast into breast cancer, which is complementary to the mechanism of present-day breast cancer development. It showed that CD3^+^T cells, CD4^+^T cells, CD8^+^T cells, CD4^+^/CD8^+^ ratio, DNT cells, NK cells, and LMR were significantly lower in the breast cancer group than in the group with benign breast lesions. In patients with lymph node metastasis and high Ki-67 expression, CD3^+^T cells, CD4^+^T cells, CD8^+^T cells or LMR appeared to be reduced to different degrees, suggesting that immunosuppression and chronic inflammation also have a promotional role in the proliferation of tumor cells and the escape of tumors from the primary site. Moreover, in clinically advanced patients, CD4^+^/CD8^+^ ratio and LMR decreased, while CD8^+^T cells levels increased, indicating that different immune cells within the CD8^+^T cells may play an inconsistent role at different stages of tumor progression. Suppressive CD8^+^T cells will dominate when the tumor progresses to advanced stages, which will exacerbate the already existing immunosuppression. HER-2 (+) patients were found to exhibit prominent immune suppression, characterized by lower levels of CD3^+^T, CD4^+^T, and CD8^+^T cells. This may be one of the reasons why patients with HER-2 overexpression have a more aggressive tumor status. Combining immunotherapy with blockade of HER-2 pathway may enhance treatment efficacy in these patients. However, the study found no significant correlation between the expression levels of lymphocyte subsets and LMR and histologic grade and pathology type. Additionally, CD19^+^B cells were not found to have a significant correlation with breast cancer in this study. Further exploration on the role of CD19^+^B cells in breast cancer can be conducted by starting from their mechanism in immune function and expanding the sample size. In conclusion, serum lymphocyte subsets and LMR are valuable for the early diagnosis of breast cancer and can provide insight into certain clinicopathological features of breast cancer. Therefore, the relationship between these factors and tumor characteristics in breast cancer patients deserves further study. By doing so, we can better understand the connection between breast cancer, the immune function of the body, and chronic inflammatory state. This knowledge may lead to the identification of brand-new therapeutic targets and offer alternative treatment options beyond the conventional approaches for breast cancer.

## Data Availability

The data supporting the findings of this study are available from the corresponding author upon reasonable request.
